# Redifferentiation Effect of Larotrectinib for *NTRK* Fusion-Positive Pediatric Thyroid Cancer and Outcomes After Therapy

**DOI:** 10.1210/jendso/bvaf098

**Published:** 2025-06-05

**Authors:** Luz E Castellanos, Sireesha Yedururi, Steven G Waguespack

**Affiliations:** Department of Endocrine Neoplasia and Hormonal Disorders and Department of Pediatrics-Patient Care, The University of Texas MD Anderson Cancer Center, Houston, TX 77030, USA; Department of Abdominal Imaging, The University of Texas MD Anderson Cancer Center, Houston, TX 77030, USA; Department of Endocrine Neoplasia and Hormonal Disorders and Department of Pediatrics-Patient Care, The University of Texas MD Anderson Cancer Center, Houston, TX 77030, USA

**Keywords:** childhood thyroid cancer, lung metastases, radioactive iodine, TRK fusion, drug holiday

## Abstract

**Context:**

Few data exist regarding larotrectinib therapy in pediatric *NTRK* fusion–positive papillary thyroid cancer (PTC), especially its effects on redifferentiation in radioactive iodine–refractory (RAIR) disease.

**Objective:**

To describe redifferentiation effects and disease outcomes in patients with stage 2 pediatric PTC following treatment with larotrectinib ± RAI.

**Methods:**

A retrospective case series at a tertiary cancer center of patients with *NTRK* fusion–positive pediatric PTC and RAIR pulmonary metastases treated with larotrectinib and considered for ^131^I therapy. Tumor response was assessed utilizing RECIST 1.1.

**Results:**

Four patients (aged 6-16 years at PTC diagnosis; 50% female) were treated with larotrectinib 100 mg twice a day for a median of 14 months (range 6-30 months). Treatment was well tolerated, except for grade 3 hypocalcemia in 1 patient with pre-existing hypoparathyroidism. All patients had tumor shrinkage (−25% to −100%) in target and nontarget pulmonary metastases. On diagnostic ^123^I thyroid scans, any RAI uptake was identified in only 2 patients, and therapeutic ^131^I did not cause further incremental tumor shrinkage in the patients treated, despite robust pulmonary uptake on the post-therapy scans. After stopping larotrectinib and with a mean follow-up of 38 months (range 26-48 months), 2 patients had stable disease and 2 had clinically insignificant tumor progression.

**Conclusion:**

Although larotrectinib can have a redifferentiation effect in pediatric *NTRK* fusion-positive PTC, therapy with ^131^I may not lead to an incremental benefit in established RAIR disease. Significant structural disease progression did not occur after cessation of larotrectinib, suggesting that a drug holiday can safely be considered in this population.

Children with papillary thyroid cancer (PTC) present with larger tumors and more frequent lymph node and pulmonary metastatic disease than adults [1], but even in stage 2 patients with lung metastases, long-term survival is excellent [1, 2]. Molecular drivers of pediatric PTC mostly comprise gene fusions involving *RET*, *NTRK1*, and *NTRK3* [3, 4], with TRK fusions identified in about 25% of patients at a tertiary cancer center who develop distantly metastatic disease [2]. In children with advanced TRK fusion–positive PTC, fusions involving *NTRK1* and *NTRK3* appear to be equally distributed [5]. While only needed in few pediatric patients with PTC, targeted therapies for these oncogenic gene fusions have been approved by regulatory agencies for use in children.

Stage 2 pediatric PTC has historically been treated with repeated courses of radioactive iodine (RAI), but recent studies have suggested low cure rates with this approach [2, 6-8]. Furthermore, it is increasingly recognized that RAI-refractory (RAIR) disease occurs in children, even though this remains to be formally defined in the pediatric population. RAIR disease occurs when thyroid cancer is unable to incorporate iodine at sites of disease due to decreased iodine metabolism–related gene expression from activation of mitogen-activated protein kinase (MAPK) signaling [9-11]. Recent strategies to approach this include resensitization of tumor cells to RAI by inhibition of MAPK pathway signaling, as demonstrated by the use of BRAF ± MEK inhibitors in *BRAF* V600E-mutated thyroid cancers [9, 12-14]. The degree of radioiodine uptake in metastatic PTC is related to the underlying oncogenic driver, and fusion-positive PTCs have variable RAI sensitivity related to the expression of thyroid differentiation genes [3, 11]. More recently, there has been an interest in the use of fusion-directed targeted therapy to enhance RAI uptake in RAIR disease, colloquially referred to as “redifferentiation” [3, 15].

Larotrectinib, a first in class selective TRK inhibitor, has demonstrated efficacy in advanced *NTRK* fusion–positive PTC [16], and several publications have shown the effect of larotrectinib on enhancing RAI uptake in lung metastases [3, 17-20], including 1 adolescent treated with larotrectinib followed by RAI in the neoadjuvant setting [19]. To our knowledge, no prior publication has reported the oncologic outcomes after RAI therapy in patients with RAIR PTC taking larotrectinib, nor have any data been presented on disease outcomes after discontinuation of therapy. In the present case series, we aim to address this knowledge gap by presenting a series of 4 patients with RAIR, *NTRK* fusion–positive, pediatric PTC treated with larotrectinib and considered for therapeutic RAI.

## Materials and Methods

For this retrospective case series, we used an IRB-approved, ambispective study with a waiver of informed consent (DR09-0507; A Database for the Study of the Clinical Presentation, Treatment, and Outcomes of Pediatric Thyroid Malignancies) and identified 4 consecutive patients with stage 2, RAIR pediatric PTC taking larotrectinib who underwent a diagnostic ^123^I whole-body scan and evaluated for ^131^I therapy (Table 1). Stage 2 disease was defined as enlarging pulmonary nodules or >10 noncalcified, solid pulmonary nodules predominantly in the lower lung fields coupled with a detectable thyroglobulin (Tg) or elevated and nondeclining Tg antibody (TgAb) titers or radioiodine uptake in the lungs on a whole body scan [2]. For this study, we defined RAIR disease as structural disease without RAI uptake on a previous post ^131^I treatment whole body scan, unequivocal progression of structural disease after prior ^131^I, even if the lung metastases were previously RAI-avid, or measurable metastatic disease without uptake on a diagnostic scan after prior ^131^I (Table 1). All patients’ tumors were tested with DNA- and RNA-based next-generation sequencing, which confirmed an isolated *NTRK1* or *NTRK3* fusion and no other oncogenic driver. One patient (Patient C) had been treated on a clinical trial and included in a previously published study [16], and the others were treated on label by the senior author after appropriate informed consent. Tumor response during treatment and after treatment completion was evaluated using the Response Evaluation Criteria in Solid Tumors (RECIST) guideline, version 1.1 [21]. One case (Patient B) did not have RECIST-measurable disease. Instead, measurements for 2 representative subcentimeter lesions were used to represent the response to therapy. All patients received the standard larotrectinib dose of 100 mg by mouth twice daily while maintaining a thyrotropin (TSH) level <0.1 mIU/L. All patients had or developed TgAbs, which precluded the accurate measurement of Tg (Table 2).

**Table 1.** Baseline characteristics of 4 patients with radioactive iodine–refractory, *NTRK* fusion–positive pediatric PTC treated with larotrectinib

Patient	Age at PTC diagnosis	Sex	AJCC8 staging	*NTRK* fusion	Cumulative RAI dose before larotrectinib	Reason PTC was considered RAIR
A	6 y	F	T3N1bM1	*IRF2BP2*::*NTRK1*	153 mCi (5.7 GBq)	Structural disease progression despite 2 ^131^I courses; negative Dx scans with macroscopic lung metastases
B	10 y	F	T3N1bM1	*ETV6*::*NTRK3*	100 mCi (3.7 GBq)	No RAI uptake in macroscopic lung metastases on post-treatment scan
C	9 y	M	T3N1bM1	*IRF2BP2*::*NTRK1*	382 mCi (14.1 GBq)	Structural disease progression despite 2 ^131^I courses and previously RAI-avid lung disease
D	16 y	M	T4aN1bM1	*TPR*::*NTRK1*	148 mCi (5.5 GBq)	Structural disease progression despite prior ^131^I; negative Dx scan with macroscopic lung metastases

Abbreviations: AJCC8, American Joint Committee on Cancer Staging Manual, 8th edition; Dx, diagnostic; F, female; M, male; *NTRK*, neurotrophic tyrosine receptor kinase; PTC, papillary thyroid carcinoma; RAI, radioactive iodine; RAIR, radioactive iodine refractory; y, years.

**Table 2.** Timeline and clinical data of 4 patients with radioactive iodine–refractory, *NTRK* fusion–positive pediatric PTC treated with larotrectinib

Patient	Time from diagnosis to laro start	Age at laro start	Baseline Tg*^a^*	Baseline TgAb*^a^*	Duration of laro Rx	Age at Dx ^123^I scan on laro	sTg*^a^* day of Dx ^123^I scan	TgAb*^a^* day of Dx ^123^I scan	Time from end of laro to last restaging	Tg*^a^* at last FU	TgAb*^a^* at last FU
A	123 mo	16 y	1.81	2164.8	15 mo	17 y	31.66	341.7	37 mo	1.19	1048.7
B	27 mo	13 y	<0.1	4865	6 mo	13 y	1.46	457.5	41 mo	<0.1	261.7
C	50 mo	13 y	213*^b^*	<20*^b^*	30 mo	16 y	163.82	7.0	48 mo	192.45	8.1
D	51 mo	20 y	0.69	8068.1	13 mo	22 y	2.96	681.7	26 mo	0.35	723.9

Abbreviations: Dx, diagnostic; FU, follow-up; laro, larotrectinib; mo, months; Rx, treatment; (s)Tg, (stimulated) thyroglobulin; TgAb, thyroglobulin antibody; y, years.

^a^Reference ranges: Tg <0.1 ng/mL for an athyrotic patient, TgAb <3.9 IU/mL.

^b^Reference ranges for this data only: Tg <0.9 ng/mL for an athyrotic patient, TgAb <40 IU/mL.

## Results

### Patient A

**Figure 1.**
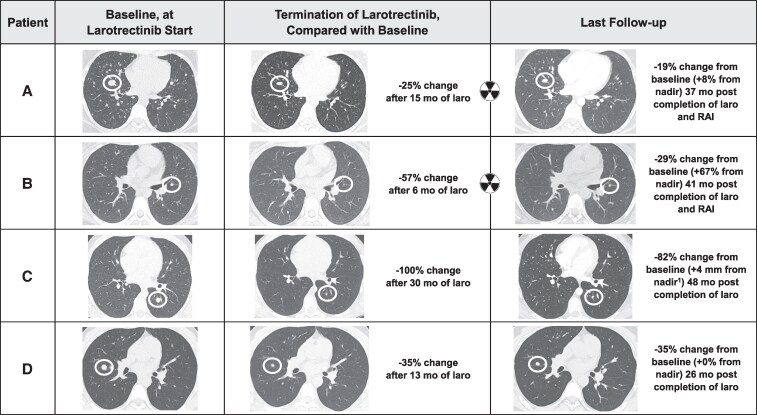
Radiographic findings in 4 patients treated with larotrectinib for stage 2, radioiodine iodine-refractory, *NTRK* fusion-positive pediatric PTC. Axial computed tomography images of lung target lesions (white circles) at start and termination of larotrectinib as well as at last follow-up after completion of therapy, including radioactive iodine (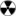) in Patients A and B. laro, larotrectinib; mo, months; RAI, radioactive iodine. ^1^Nadir response in the sum of the target lesion diameters was 0 mm and thus a percentage increase from nadir was incalculable.

**Figure 2.**
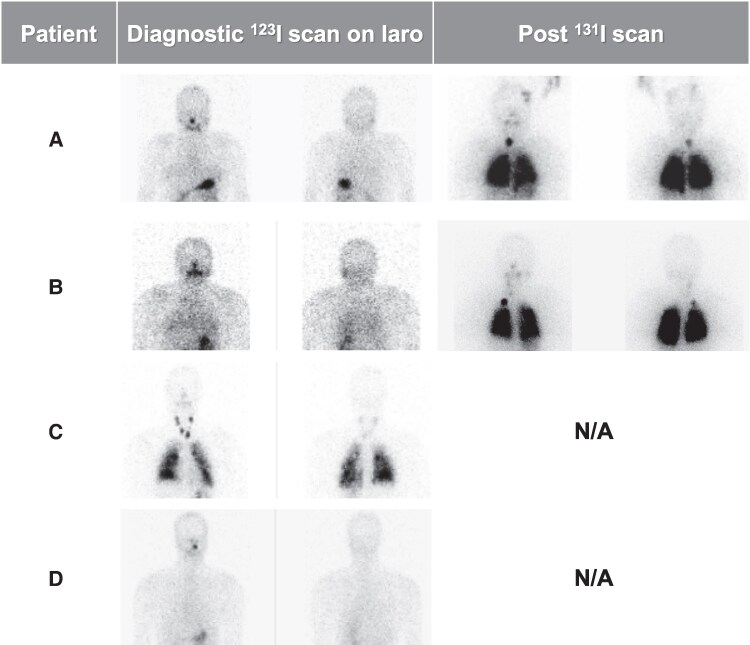
Nuclear scintigraphic findings in 4 patients treated with larotrectinib for stage 2, radioiodine iodine-refractory, *NTRK* fusion–positive pediatric PTC. Planar images from diagnostic ^123^I and post-therapy thyroid scans at termination of larotrectinib and after administration of ^131^I (if applicable), respectively. Each image includes anterior (left) and posterior (right) views. Abbreviations: laro, larotrectinib; N/A, not applicable.

**Figure 3.**
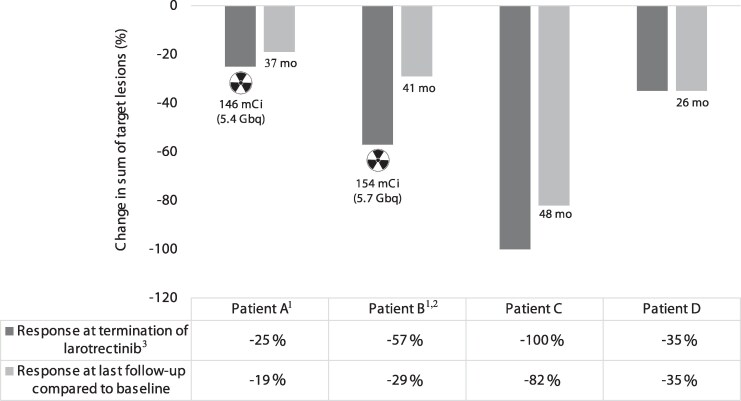
RECIST evaluation in 4 patients with stage 2, radioactive iodine–refractory, pediatric NTRK fusion–positive PTC treated with larotrectinib ± radioactive iodine. Measurements show the percent change in the sum of the target lesion diameters before stopping larotrectinib and the response, compared with baseline, at the last staging visit while on active surveillance and TSH suppression. ^1^Patients A and B received therapeutic ^131^I (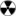) at termination of larotrectinib. ^2^Patient B did not have RECIST measurable target lesions. ^3^This also equaled the nadir response.

Diagnosed at age 6 years with a T3bN1bM1 PTC, Patient A had RAIR lung metastases based on negative diagnostic scans and pulmonary progression despite 2 ^131^I courses. She had previously been treated from age 10-12 years with sorafenib. Due to RECIST-measurable progression in the lungs, she started larotrectinib at the age of 16 years. Side effects included transient, mild nonspecific gastrointestinal complaints; she subsequently experienced no further adverse events. After 15 months of treatment and a best response of stable disease (SD) (25% decrease in the sum of the target lesion diameters and a non-complete response [CR]/non-progressive disease [PD] in nontarget lesions) (Fig. 1), the patient underwent a ^123^I diagnostic scan that showed no unequivocal pulmonary uptake. She was empirically administered 146 mCi (5.4 GBq) ^131^I with a post-treatment scan showing right thyroid bed and significant bilateral pulmonary uptake (Fig. 2). Larotrectinib was stopped the morning of RAI therapy. Thirty-seven months after RAI and cessation of larotrectinib, there was continued SD in the lung metastases by RECIST 1.1. The sum of target lesion diameters increased minimally in size (+8% from nadir) (Figs. 1 and 3), and the nontarget lesions did not show unequivocal progression.

### Patient B

Patient B was diagnosed with PTC (T3aN1bM0) at age 10 years. She had no uptake on a post-treatment scan after receiving 100 mCi (3.7 GBq) ^131^I, and multiple subcentimeter pulmonary metastases were identified 13 months after RAI when she had her initial computed tomography scan of the chest. She started larotrectinib at age 13 years due to progression of pulmonary disease over a 6-month period with the goal of reducing the volume of disease followed by a tentative second treatment with ^131^I. The subcentimeter lung metastases did not meet the size criteria for measurable/target lesions by RECIST 1.1. She tolerated larotrectinib without any adverse effects, and the nontarget lung metastatic lesions showed a non-CR/non-PD. Two representative lung metastases were measured, and these showed a 57% decrease in the sum of their diameters (Fig. 1). After a ^123^I diagnostic scan showed diffuse low-level activity in both lungs, she received 154 mCi (5.7 GBq) ^131^I with the post-treatment scan showing diffuse and intense bilateral lung uptake, in addition to uptake in a right supraclavicular lymph node. (Fig. 2) Larotrectinib was stopped the morning before RAI therapy, and she suffered myalgias, as has been well described after larotrectinib withdrawal. The pulmonary metastatic disease did not show any further decrease in size after RAI therapy and, 41 months after cessation of larotrectinib and RAI, there was a 67% increase from nadir in the sum of the 2 representative lesions (6-10 mm, non-CR/non-PD per RECIST; considered clinically insignificant) (Figs. 1 and 3).

### Patient C

Diagnosed with a T3bN1bM1 at age 9 years and found to have innumerable miliary metastases in both lungs, Patient C had RAI-avid disease that was considered refractory due to RECIST progression despite a cumulative administered activity of 382 mCi (14.1 GBq) ^131^I. Due to progressive pulmonary disease, including new lesions over the course of 19 months, he started larotrectinib at age 13 years on a clinical trial and had a partial response (PR) overall (CR [−100%] in the 2 target lesions and a non-CR/non-PD in nontarget lesions) (Fig. 1), as previously reported [16]. He experienced multiple adverse effects, including grade 3 hypocalcemia (postoperative hypoparathyroidism present at baseline) and grade 1 fatigue, arthralgias, dizziness, and lab abnormalities (leukopenia, elevated creatinine, elevated uric acid). After 30 months of larotrectinib therapy, a diagnostic thyroid scan demonstrated significant cervical and lung uptake (Fig. 2), which was notably more intense than the prior post-treatment scan (not shown). ^131^I was not given due to the excellent structural response, the already high cumulative ^131^I activity, and the shared decision to stop systemic therapy and start active surveillance. Following 2 years of stability, there was a minimal increase in the sum of the target pulmonary metastases (0-4 mm) and minimal PD in nontarget lesions 36 and 48 months after cessation of larotrectinib (Figs. 1 and 3). The pulmonary disease progression was not considered clinically significant and he continues on TSH suppressive therapy and active surveillance.

### Patient D

Diagnosed with PTC (T4aN1bM1) at age 16 years with innumerable pulmonary metastases, Patient D initially had RAI-avid disease treated with 147 mCi (5.4 GBq) ^131^I. RECIST progression occurred despite this, and his diagnostic thyroid scan was negative even with lung metastases measuring up to 10.5 mm. He started larotrectinib at age 20 years. Side effects included grade 2 alanine aminotransferase elevation, grade 1 aspartate aminotransferase elevation, headache, and myalgias. After 13 months of treatment and a best response of PR (35% decrease in sum of target lesions and non-CR/non-PD in nontarget lesions, (Fig. 1) a ^123^I diagnostic scan was repeated and remained negative (Fig. 2). The mutual decision was not to treat with empiric ^131^I and to initiate active surveillance after cessation of larotrectinib. Twenty-six months after treatment cessation, he had SD with a 0% change in the target lesions from the nadir response and persistent non-CR/non-PD in nontarget lesions (Figs. 1 and 3).

## Discussion

This report is the first series of patients with RAIR pediatric *NTRK* fusion–positive PTC evaluated for the redifferentiation effects of larotrectinib, and it represents the only case series in either children or adults documenting structural disease outcomes after therapy with high-dose ^131^I and discontinuation of a selective TRK inhibitor.

Of our 4 patients undergoing a diagnostic ^123^I thyroid scan while taking larotrectinib, 1 had no evidence of RAI uptake (Patient D), 2 (Patients A and B) had no significant redifferentiation effect on the diagnostic studies but were nonetheless treated with ^131^I and had excellent pulmonary uptake seen on the post-treatment scans, and 1 had evidence for significant ^123^I uptake (Patient C) but was not treated with RAI due to his already high cumulative administered activity. These cases demonstrate the variable effects of selective TRK inhibition on RAI uptake in pediatric PTC, as has also been described in adults [18, 20]. One hypothesis for this differential response is that coexistent pathogenic variants in other genes such as *TERT* may play a role [20]. Importantly, *TERT* promoter mutations were not checked in all of our patients, but these variants are extraordinarily rare in pediatric PTC [22] and would, therefore, likely be absent. Furthermore, next-generation sequencing identified no other somatic alterations in our patients’ tumors to explain the differences in RAI avidity. The specific fusion also does not appear to predict response because, in the 2 patients whose tumors had the same fusion (Patients A and C), there were disparate responses in RAI uptake. This could be explained by the fact that Patient A was considered RAIR based on no uptake in structural disease whereas Patient C was considered RAIR due to progression of previous RAI-avid disease. Another important factor to consider regarding the variable responses to larotrectinib includes therapy duration. Our patients’ duration of systemic treatment ranged from 6 to 30 months, and whether or not the length of therapy affects RAI uptake in RAIR pediatric PTC remains to be determined.

Disappointingly, in the 2 patients treated with high activity ^131^I, there was no incremental decline in tumor volume despite excellent pulmonary uptake on the post-treatment scans. This would suggest that a redifferentiation approach may not work in established RAIR disease and could be considered earlier, such as prior to the initial dose of ^131^I in patients with significant structural disease [19]. Studies such as the recently launched LANTERN trial (NCT05783323) will help to better clarify the positioning of larotrectinib in the management of RAIR TRK-fusion PTC.

In children with stage 2 PTC who require targeted therapy, it is untenable to consider lifelong therapy, yet no data currently exist showing disease outcomes after cessation of oncogenic fusion-targeted therapy in this population. In this case series, after stopping larotrectinib ± RAI, Patients A and D had SD for 37 and 26 months, respectively, Patient C had PD about 3 years after withdrawing larotrectinib but showed SD for the first 2 years, and Patient B (who did not have RECIST-measurable disease) had minimal pulmonary disease progression. In both Patients B and C, the tumor enlargement was not considered clinically significant and their disease burden was still lower than it had been at baseline (Fig. 1). Our experience would suggest that withdrawing larotrectinib does not result in rapid tumor progression and that a prolonged drug holiday is feasible in young patients with pediatric-onset PTC. The indolent nature and excellent prognosis of stage 2 pediatric PTC [2] also support the option of active surveillance in the setting of metastatic disease, even if disease were to progress minimally after cessation of targeted therapy.

Only a single pediatric case report with follow-up of after discontinuation of larotrectinib for PTC has previously been published [19]. This patient had a *TPM3*::*NTRK1* fusion PTC and was treated with larotrectinib for 24 weeks prior to the initial administration of ^131^I. The baseline diagnostic ^123^I scan showed faint uptake in the pulmonary disease without significant uptake noted in the largest metastatic lesions. Repeat diagnostic scan at termination of larotrectinib showed significantly improved RAI uptake, including in sites of previous nonavid disease. Follow-up 12 months off larotrectinib and ^131^I showed significant radiographic responses in all metastatic lesions and a decline in Tg levels. At this patient's last visit, now 5 years after diagnosis and 50 months after therapeutic RAI, she has an indeterminate response to therapy with unchanged, scattered punctate pulmonary nodules <4 mm and a suppressed Tg of 0.15 ng/mL (<0.1 for an athyrotic individual) with negative TgAb (S.G.W. personal communication).

Withdrawal of larotrectinib has also been successful in select pediatric patients with *NTRK* fusion–positive sarcomas and related mesenchymal tumors, as recently reported in a global multicenter trial data set that included 47 patients who discontinued larotrectinib [23]. After a median follow up of 41 months, the median time to progression was not reached (range 0+ to 77.6+ months), and in the 16 patients who progressed/relapsed during active surveillance, 69% had an objective response to larotrectinib rechallenge. Although none of our patients required re-initiation of larotrectinib for disease progression after a mean follow up of 38 months, we would presume that re-initiation of larotrectinib (if needed for clinically significant disease progression) would be effective based on the study by Mascarenhas et al [23].

In conclusion, in this small single institution case series, there are variable redifferentiation effects of larotrectinib treatment in adolescent and young adult patients with *NTRK* fusion–positive pediatric PTC, and therapeutic ^131^I after larotrectinib in established RAIR disease did not result in additional structural response. In patients with stage 2 pediatric PTC requiring larotrectinib, cessation of targeted therapy is not associated with clinically relevant disease progression after over 3 years of follow-up. Further studies are needed to define the optimal management strategy for patients with pediatric *NTRK* fusion-positive PTC and pulmonary metastases.

## Data Availability

Original data generated and analyzed during this study are included in this published article or in the data repositories listed in References.

## Abbreviations

CR: complete response
MAPK: mitogen-activated protein kinase
PD: progressive disease
PR: partial response
PTC: papillary thyroid cancer
RAI: radioactive iodine
RAIR: radioactive iodine refractory
SD: stable disease
Tg: thyroglobulin
TgAb: thyroglobulin antibody
TSH: thyrotropin
